# Hyper-Osmotic Stress Elicits Membrane Depolarization and Decreased Permeability in Halotolerant Marine *Debaryomyces hansenii* Strains and in *Saccharomyces cerevisiae*

**DOI:** 10.3389/fmicb.2019.00064

**Published:** 2019-01-29

**Authors:** Claudia Capusoni, Stefania Arioli, Silvia Donzella, Benedetta Guidi, Immacolata Serra, Concetta Compagno

**Affiliations:** Department of Food, Environmental and Nutritional Sciences, University of Milan, Milan, Italy

**Keywords:** marine microorganisms, *Debaryomyces hansenii*, *Saccharomyces cerevisiae*, osmotic stress, flow cytometry, membrane depolarization, membrane permeability, bioprocesses

## Abstract

The use of seawater and marine microorganisms can represent a sustainable alternative to avoid large consumption of freshwater performing industrial bioprocesses. *Debaryomyces hansenii*, which is a known halotolerant yeast, possess metabolic traits appealing for developing such processes. For this purpose, we studied salt stress exposure of two *D. hansenii* strains isolated from marine fauna. We found that the presence of sea salts during the cultivation results in a slight decrease of biomass yields. Nevertheless, higher concentration of NaCl (2 M) negatively affects other growth parameters, like growth rate and glucose consumption rate. To maintain an isosmotic condition, the cells accumulate glycerol as compatible solute. Flow cytometry analysis revealed that the osmotic adaptation causes a reduced cellular permeability to cell-permeant dye SYBR Green I. We demonstrate that this fast and reversible phenomenon is correlated to the induction of membrane depolarization, and occurred even in presence of high concentration of sorbitol. The decrease of membrane permeability induced by osmotic stress confers to *D. hansenii* resistance to cationic drugs like Hygromycin B. In addition, we describe that also in *Saccharomyces cerevisiae* the exposure to hyper-osmotic conditions induced membrane depolarization and reduced the membrane permeability. These aspects are very relevant for the optimization of industrial bioprocesses, as in the case of fermentations and bioconversions carried out by using media/buffers containing high nutrients/salts concentrations. Indeed, an efficient transport of molecules (nutrients, substrates, and products) is the prerequisite for an efficient cellular performance, and ultimately for the efficiency of the industrial process.

## Introduction

The use of microbial cells for industrial bio-productions has been promoting the transition to green chemistry for the synthesis of molecules by means of more eco-compatible processes. The isolation and study of microorganisms with useful properties is therefore a fundamental step in the development of such processes. At industrial level, microbial cells are often cultivated under conditions that can exert stress. Salts (ionic) and sugars/polyols (non-ionic) are some of the principal constituents of cultural media and represent the principal osmotic stressors when used at high concentration as in several fermentation processes. High salt levels are present in the second-generation bioethanol production process, due to the pretreatments used to detoxify the hydrolysate from lignocellulosic materials (Sanchez and Cardona, 2008). These stressful conditions compromise the cellular performance resulting in decreased production efficiency. In this regard, adequate stress tolerance of the industrial strains has turned out to be a major challenge for obtaining economically sustainable production (Deparis et al., 2017). Recently, marine yeasts have been explored with the aim to develop industrial bioprocesses employing seaweeds as carbon source as well as seawater, which can represent a sustainable alternative to the large consumption of freshwater (Domínguez de María, 2013; Zaky et al., 2014; Zambelli et al., 2015; Serra et al., 2016). Their industrial potential as biocatalysts is increasing also, due to the strict relation among salt and organic solvent tolerances often observed for enzymes obtained from halotolerant species (Trincone, 2011). Therefore, the understanding of adaptive responses triggered to maintain proper cell homeostasis under hyperosmotic conditions is a priority for the improvement of such biotechnological processes.

Yeast species exhibit different levels of osmotic tolerance, and the studies on the cellular mechanisms behind highlight how yeasts employ different strategies to survive (Ramos et al., 2011; Bubnová et al., 2014; Dakal et al., 2014; Mukherjee et al., 2017). In *Saccharomyces cerevisiae*, the osmotic response has been widely explored due to its high biotechnological importance and the broad availability of analytical methods. High osmolality activates the HOG-MAPK signaling cascade (Hohmann, 2015), resulting in adaptive responses such as accumulation of glycerol. Structural properties of the cell wall and plasma membrane are important factors influencing the yeast osmotolerance (Levin, 2011). *S. cerevisiae* has been reported to shorten the fatty-acid chain length, and to increase their saturation level upon the rise of salinity (Turk et al., 2007). Membrane potential is generated by ions gradients across the membranes of living cells, and it is obviously affected by salt-induced osmotic stress. Plasma membrane depolarization caused by NaCl has been observed in *S. cerevisiae*, and predicted by mathematical models of ion fluxes regulation (Ke et al., 2013; Plášek et al., 2013).

Osmotic response has been studied in few halotolerant species, like *Debaryomyces hansenii* (Prista et al., 2005). Detailed researches on Na^+^ and K^+^ movements during osmotic stress and on expression of genes encoding the ion transporters/exchangers demonstrated the important role played by these mechanisms (Almagro et al., 2001; González-Hernández et al., 2004; Velkova and Sychrova, 2006; Michan et al., 2013). Recently, a mechanism that entails the sequestration of surplus Na^+^ cations in intracellular compartments has been reported (Herrera et al., 2017), confirming its previously reported attitude as “Na-includer” yeast.

In the present work, we have investigated aspects related to the osmotic adaptation in marine yeasts collected at a deep sea hydrothermal vent (South Pacific West, Lau Basin, 2620 m below sea surface level) (Burgaud et al., 2010). In particular, two *D. hansenii* strains, isolated from gasteropod (*Ifremeria nautilei*) gills and from coral, showed halotolerance and were studied in detail. *D. hansenii* possesses metabolic traits that look appealing for developing industrial processes (Prista et al., 2016). Strains associated with cheese and meat processing, have been reported to contribute to their final aroma and, by the activity of particular proteolytic enzymes, to alter food composition (Gori et al., 2012). Strains isolated from cheese and fish gut have been recently investigated for potential probiotic properties (Ochangco et al., 2016). Other examples of its biotechnological interest are its usage for biocontrol of ochratoxigenic molds (Iacumin et al., 2017), production of enzymes like exopeptidases and thermophilic β-glucosidases, production of fine chemicals, such as xylitol and riboflavin as well as for its ability to use a broad spectrum of carbon substrates (Breuer and Harms, 2006). Considering the potential for applications of this species, there is an interest for the development of sustainable industrial bioprocesses.

By using flow cytometry, a methodology that rapidly allows obtaining accurate information regarding important cellular parameters at single cell level, monitoring in this way the heterogeneity of the cellular population (Comas-Riu and Rius, 2009; Müller and Nebe-von-Caron, 2010), we describe that membrane depolarization and decrease of membrane permeability reversibly occur upon hyper-osmotic stress in *D. hansenii* strains. The decrease of membrane permeability confers to *D. hansenii* resistance to cationic drugs like Hygromycin B. In addition, we show that these osmotic responses are induced also in less osmotolerant species like *S. cerevisiae*. These effects are relevant for the development of industrial bioprocesses, particularly in the case of seawater-based processes. High concentrations of nutrients and salts in cultivation media as well as in bioconversion buffers exert in fact a strong osmotic pressure, in this way influencing the efficiency of solute transport (nutrients, substrates, and products) and, in turn, the efficiency of the whole process.

## Materials and Methods

### Strains and Growth Conditions

The strains used in this work belong to a collection of marine yeasts created previously from deep sub-seafloor sediments and deep-sea hydrothermal vents (Burgaud et al., 2010). All isolates are available at the UBO Culture Collection^1^.

For long-term storage, yeast strains, including *S. cerevisiae* strain CENPK113-7D, were maintained at −80°C on 15% (v/v) glycerol and 85% (v/v) YPD (10 g/l yeast extract, 20 g/l peptone and 20 g/l glucose).

The screening in presence of NaCl was performed on plates containing defined minimal medium Yeast Nitrogen Base (YNB, Difco, Italy) containing glucose 2% (w/v, Sigma-Aldrich, Italy), 2% agar (w/v, Conda, Spain), supplemented with 2-(N-Morpholino) Ethane Sulfonic acid (MES, Sigma-Aldrich, Italy) 0.1 M at pH 6, and containing different concentrations of NaCl, ranging from 0.5 to 2 M.

Broth cultures were performed at 28°C in flasks under shaking conditions at 150 rpm (INFORS HT, Multitron Standard). Cells from pre-cultures grown in YNB-glucose-MES were harvested during the exponential growth phase by centrifugation and inoculated at OD_600*nm*_ 0.1 into the same medium supplemented with 4% (w/v) sea salts (SS, Sigma-Aldrich, Italy), or 2 M NaCl, or 2 M sorbitol (Sigma-Aldrich, Italy), or not supplemented (control cultures). The growth was monitored through the increase in OD at 600_nm_ using a spectrophotometer (Jenway, 7315^TM^ Bibby Scientific Limited, Stone, United Kingdom). Broth cultures were performed in triplicate.

In *D. hansenii* to apply hyper-osmotic shock, cells growing in YNB-glucose-MES (control condition) were harvested during the exponential growth phase, centrifuged at 2,300 *g* for 5 min and shifted to flasks containing YNB-glucose-MES supplemented with 4% (w/v) SS (naturally containing 0.55 M NaCl), 2 M NaCl, for 30 min up to 2 h, or 2 M sorbitol and incubated for 2 h. In *S. cerevisiae* to apply hyper-osmotic shock, cells growing in YNB-glucose-MES (control condition) were harvested during the exponential growth phase, centrifuged 2,300 *g* for 5 min (Eppendorf 5415D) and shifted to flasks containing YNB-glucose-MES supplemented with 0.55 M NaCl for 2 h.

For hypo-osmotic shock, cells growing in YNB-glucose-MES in presence of 4% (w/v) SS or 2 M NaCl were incubated in the same medium without salts for 2 h.

The effect of hyper- and hypo-osmotic stress was assessed by flow cytometry (FCM) by evaluating cell membrane permeability and cell membrane potential (see below).

For testing cationic drug sensitivity after exposition toward salts, aliquots of tenfold serial dilutions were spotted on YNB-glusose-MES plates supplemented or not with 2 M NaCl and in presence of increasing concentrations of Hygromycin B (10, 25, and 75 μg/ml) (Sigma-Aldrich, Italy). Then the plates were incubated at 28°C for 3 days.

### Dry Weight and Metabolite Assays

For dry weight measurements (DW), samples from different culture conditions were collected at different times (in triplicate at each point). Cells were filtered through a glass microfiber GF/A filter (Whatman), washed with three volumes of de-ionized water and dried at 100°C for 24 h.

Glucose, ethanol, and glycerol concentrations in the supernatants of the cell cultures were assayed by using commercial enzymatic kits (Hoffmann La Roche, Basel, Switzerland).

For intracellular glycerol determination, cells (30 OD) were collected by centrifugation and suspended in 500 μl of water, boiled at 100°C for 15 min, then centrifuged at 16,100 *g* for 5 min, and the supernatants were assayed by using the commercial enzymatic kit (see above).

For all the assays, the standard deviation was lower than 5%.

Biomass yields and specific glucose consumption rates have been calculated according to van Hoek et al. (2000).

### FCM Analysis

SYBR Green I, propidium iodide (PI), 5(6-)-carboxyfluorescein diacetate N-succinimidyl ester (cFSE), and DiBAC_4_(3) were obtained from Sigma-Aldrich (Italy). SYTO^TM^ 24 was purchased from Thermo Fisher Scientific (Milan, Italy).

Cells were stained with SYBR Green I 1X or with SYTO^TM^ 24 5 μM at 25°C for 15 min in the dark. For the evaluation of membrane integrity or cell viability the cells were stained with PI 7.5 μM or with cFSE 5 μM and incubated in the dark for 15 min before measurement. For the evaluation of membrane depolarization, the cells were stained with DiBAC_4_(3) 5.7 μM and incubated in dark for 15 min before measurement. Briefly, cells were harvested by centrifugation (2,300 *g* 5 min) during early exponential growth phase and suspended at 10^6^ cells/ml in YNB-glucose-MES pH 6, or in the same medium supplemented with SS or 2 M NaCl. The dyes were then added at the appropriate final concentrations, and incubated as required. To cause membrane depolarization, cells growing in YNB medium were exposed for 15 min to 1 μM carbonyl cyanide 3-chlorophenylhydrazone (CCCP, Sigma-Aldrich, Italy) and then stained as described above. Cell count and fluorescence detection were performed using an Accuri C6 flow cytometer (BD Biosciences, Milan, Italy). Cell suspensions were analyzed using the FCM with the following threshold settings: FSC 5,000, SSC 4,000, and 20,000 total events collected. All the parameters were collected as logarithmic signals and 488 nm laser was used to measure the FSC values. When necessary, samples were diluted in filtered fresh media just before measurement, so that the rate of events in the flow was generally lower than 2,000 events/s. The data were analyzed using BD Accuri^TM^ C6 software version 1.0 (BD Biosciences, Milan, Italy). The SYBR Green I, SYTO^TM^ 24, cFSE and DiBAC_4_(3) fluorescence intensities of stained cells were recovered in the FL1 channel (excitation 488 nm, emission filter 530/30 nm, BD Biosciences, Milan, Italy). The PI fluorescence was recovered in the FL3 (excitation 488 nm, emission filter 610/20 nm, BD Bioscience, Milan, Italy).

## Results

### Impact of Sea Salts and 2 M NaCl on *D. hansenii* Growth Parameters

A screening performed on solid medium containing different NaCl concentrations (range 0.5–2 M) was performed in order to test the salt tolerance of yeasts isolated from endemic animal fauna living at deep-sea hydrothermal vents (Burgaud et al., 2010). This analysis revealed that *D. hansenii* was among the most tolerant species (Supplementary Table S1).

In order to study the effects of SS or higher NaCl concentration on the growth parameters, we performed broth cultures of *D. hansenii* Bio2 and Mo40 strains. The cultures were carried out under aerobic conditions, on defined minimal medium YNB containing: (i) 4% (w/v) SS, the concentration in seawater (containing 0.55 M NaCl) or (ii) 2 M NaCl, which exerts a stronger osmotic pressure.

**FIGURE 1:**
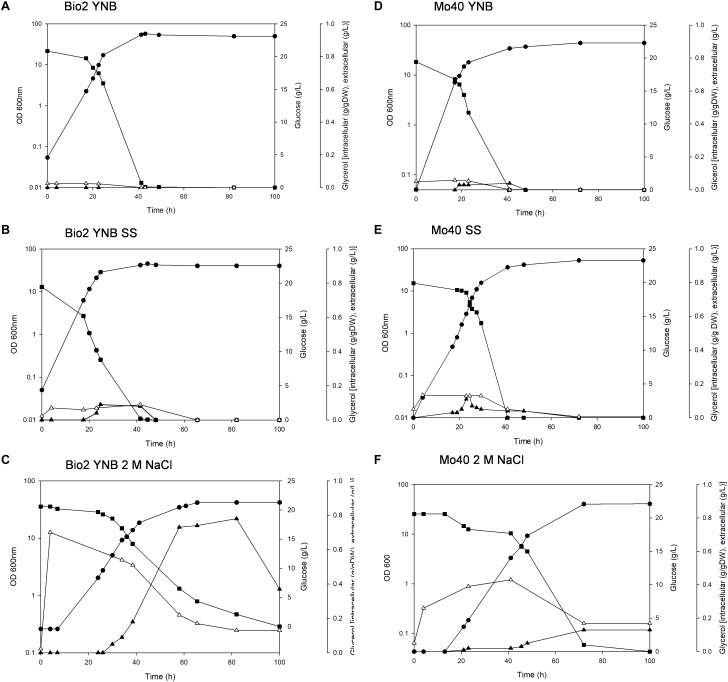
Growth of *Debaryomyces hansenii* Bio2 and Mo40 strains under different conditions. **(A,D)** Growth on YNB, control condition; **(B,E)** growth in presence of sea salts (SS); **(C,F)** growth in presence of 2 M NaCl. 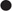
OD 600 nm, 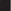
glucose (g/l), 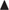
extracellular glycerol (g/l), Δintracellular glycerol (g/g of dry weight). Broth cultures were performed in triplicate and standard deviations were lower than 5%.

The presence of SS in the growth medium had a faint effect on the growth of both strains: their specific growth rates and specific glucose consumption rates were similar to those of respective control cultures (without salts) (Table 1 and Figure 1). On the contrary, higher salt concentration (NaCl 2 M) slowed down the growth, with reductions of their respective growth rates of 42 and 43% for Bio2 and Mo40, respectively (Table 1). Slower specific glucose consumption rates were also detected, with decreases of 23 and 24% for Bio2 and Mo40, respectively. The growth kinetics showed an adaptation phase lasting at least 8 h for Bio2 and 13 h for Mo40 (Figure 1C,F).

**Table 1:** Growth parameters of *Debaryomyces hansenii* Bio2 and Mo40 cultivated in presence of sea salts (SS) and 2 M NaCl.

	Bio 2				Mo40			
	μ_max_ [h^−1^]	Biomass yield (Y) [g_dw_/g_glc_]	q Glucose [mmol_glc_/g_dw_ /h]	Final biomass g/l	μ_max_ [h^−1^]	Biomass yield (Y) [g_dw_/g_glc_]	q Glucose [mmol_glc_/g_dw_ /h]	Final biomass g/l
YNB	0.26 ± 0.01	0.42 ± 0.02	2.52 ± 0.03	8.88 ± 0.24	0.28 ± 0.01	0.39 ± 0.01	2.71 ± 0.13	7.71 ± 0.14
YNB SS	0.26 ± 0.01	0.36 ± 0.01	2.56 ± 0.04	7.45 ± 0.64	0.31 ± 0.01	0.37 ± 0.01	2.59 ± 0.13	7.90 ± 0.19
YNB 2 M NaCl	0.15 ± 0.02	0.37 ± 0.01	1.95 ± 0.13	7.40 ± 0.14	0.16 ± 0.04	0.34 ± 0.02	2.05 ± 0.11	6.61 ± 0.16

The cultivation in presence of either SS or 2 M NaCl resulted in 13–14% lower biomass yields (Table 1), although the cells showed a respiratory metabolism (no production of ethanol was found in all the cultures). The discrepancy between respiratory metabolism and low biomass yield can reflect a higher amount of ATP to be redirected from the biomass synthesis toward the mechanisms of cation extrusion.

In parallel, we measured the intracellular glycerol accumulated during the growth in presence of SS and NaCl. In Bio2 and Mo40 glycerol was maintained at higher intracellular level in presence of 2 M NaCl than in sea salts, and this level decreased when the growth approached the stationary phase, concomitantly with its leakage into the medium (Figure 1C,F).

In conclusion, our results indicated that the presence of SS during the cultivation of *D. hansenii* marine strains produces only a limited decrease of the biomass yields. Nevertheless, a higher salt concentration (2 M NaCl) exerts greater negative impact on the other growth parameters (growth rate and glucose consumption rate).

### Effects of Growth Conditions on Cell Volume, Membrane Permeability and Membrane Potential

*Debaryomyces hansenii* Bio2 and Mo40 cells cultivated on media containing salts were analyzed by FCM. Bio2 exhibited a reduced cell volume in terms of forward light scattering (FSC), namely a 18 and 23% of volume decreasing when the cells were cultivated in presence of SS or 2 M NaCl, respectively. This effect was not evident in Mo40 cells cultivated in presence of salts (Supplementary Figure S1).

**FIGURE 2:**
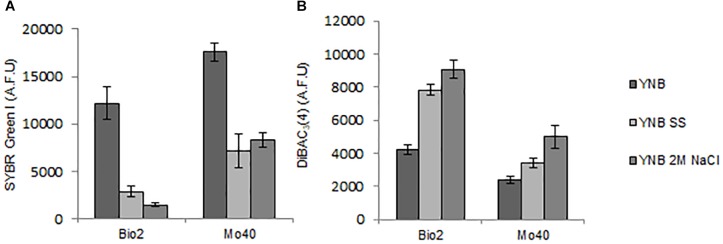
Quantitative analysis of **(A)** SYBR Green I and **(B)** DiBAC_4_(3) fluorescences detected by FMC in cells collected in exponential phase of growth on YNB (control condition), in presence of SS or 2 M NaCl. AFU, Arbitrary Fluorescence Units.

Membrane-permeant dyes that specifically bind to nucleic acids, like SYBR Green I or SYTO^TM^ 24, are usually employed to stain cells (Müller and Nebe-von-Caron, 2010). On the contrary, intact membranes of viable cells exclude PI, which is used to monitor membrane damages or cell death (Davey and Hexley, 2011). Samples of Bio2 and Mo40 exponentially growing cells cultivated on YNB without salts (control cultures) or on YNB in presence of SS or 2 M NaCl were collected and stained by maintaining the cells in the same conditions of the growth. In comparison with control cultures, cells grown on salts-containing media (therefore exposed to hyper-osmotic stress during their growth) exhibited lower levels of SYBR Green I (Figure 2A and Supplementary Figure S2). For Bio2 strain, the fluorescence decrease was of 76% on SS and 88% on 2 M NaCl. For Mo40 the decrease was of 60% on SS and 53% on 2 M NaCl (Figure 2A). On the other hand, PI stained only 1–2% of cells under all the growth conditions, indicating that most of the cells were viable and with intact membrane. To confirm the reduced membrane permeability toward permeant dyes due to the hyper-osmotic stress, Bio2 cells were stained with SYTO^TM^ 24, another cell permeant nucleic acid staining. As figured out for SYBR Green I, we measured a decreasing of fluorescence when cells were cultivated in presence of salts (Supplementary Figure S3). Moreover, when Bio2 cells growing under hyper-osmotic conditions were permeabilized by treatment with 70% ethanol their SYBR Green I fluorescence levels were equal to the control cells (Supplementary Figures S4A–C). Interestingly, if the staining was performed by diluting the cells in PBS, which is a common isotonic solution, we observed that cells growing on salts-containing media were stained at levels equal to the control cells (Supplementary Figures S4D–F). We can hypothesize that this buffer can cause a hypo-osmotic shock during the staining procedure of cells growing on salts-containing media, suggesting that this condition rapidly (30 min) restores the membrane permeability to the dye. These results indicated that the growth in presence of salts alters the cell membrane permeability.

To test if the effects observed with SYBR Green I and SYTO^TM^ 24 were exhibited also with dyes possessing different chemical characteristics, we stained Bio2 and Mo40 cells with cFSE, a lipophilic compound mainly used to assess cell viability and intracellular pH (Capusoni et al., 2016). In this case, both cells growing on media containing SS or 2 M NaCl resulted stained like the control cells (Supplementary Figure S5).

Then, we estimated membrane potential on Bio2 and Mo40 cells growing in presence of salts by staining with the oxonol dye DiBAC_4_(3), a voltage-sensitive fluorescent dye (Comas-Riu and Rius, 2009). Cells with depolarized membranes result brighter than cells with normal membrane potential (Guyot et al., 2015). Cultivation on media with salts caused membrane depolarization in both strains (Figure 2B). Specifically, for Bio2 and Mo40 cells we measured an increase of DiBAC_4_(3) fluorescence when cells were cultivated in presence of SS and NaCl. Interestingly, higher fluorescence values were detectable when cells were exposed to NaCl rather than SS during their growth (Figure 2B).

### Effect of Hyper- and Hypo-Osmotic Conditions on Membrane Permeability and Membrane Potential

**FIGURE 3:**
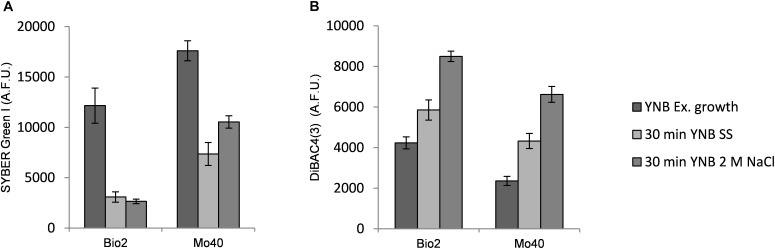
Quantitative analysis of **(A)** SYBR Green I and **(B)** DiBAC_4_(3) fluorescences detected by FMC after exposure to hyper-osmotic stress (SS or 2 M NaCl). AFU, Arbitrary Fluorescence Units.

To better understand the kinetic of the salt effect in changing membrane permeability and membrane potential, we analyzed the short-time response (from 30 min up to 2 h) toward hyper- and hypo-osmotic stress. Bio2 and Mo40 cells exponentially growing on YNB (control condition) were exposed to SS or 2 M NaCl (hyper-osmotic). By FCM we observed that 30 min of exposure to SS or 2 M NaCl were enough to determine, respectively, 75 and 78% decrease of SYBR Green I staining in Bio2 strain (Figure 3A). Similarly, in Mo40 the fluorescence decrease caused by salt stress was of 60% for SS and of 40% for 2 M NaCl (Figure 3A). After 2 h, the loss of membrane permeability in terms of reduced fluorescence was comparable to that observed in cell growing in presence of salts (not shown).

On the other hand, exposure to salts caused membrane depolarization: after 30 min an increase of DiBAC_4_(3) fluorescence of 38 and 100% was detected in Bio2 cells, and of 83 and 180% in Mo40 cells exposed to SS and 2 M NaCl, respectively (Figure 3B).

Then we investigated if hyper-osmotic conditions induced by sugars, as high concentration of sorbitol, triggered the same kind of response. The exposition of Bio2 cells to 2 M sorbitol in the growth medium resulted in 85% decrease of SYBR Green I fluorescence and generated membrane depolarization in 2 h (Supplementary Table S2). We concluded that loss of permeability and membrane depolarization are a general hyper-osmotic stress response.

**FIGURE 4:**
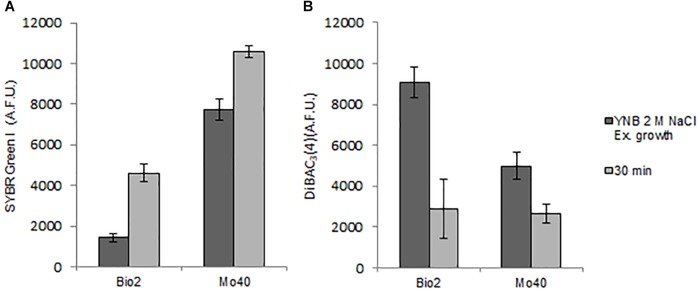
Quantitative analysis of **(A)** SYBR Green I and **(B)** DiBAC_4_(3) fluorescences detected by FMC after exposure to hypo-osmotic stress for 30 min. AFU, Arbitrary Fluorescence Units.

In order to test if the shift to hypo-osmotic conditions restored membrane permeability and membrane polarization, Bio2 and Mo40 cells cultivated in presence of 2 M NaCl were stained in YNB without salt. The FCM analysis showed that after 30 min an increase of SYBR Green I fluorescence was already visible in both strains (Figure 4A), concomitantly with the process of membrane repolarization, detected by the decrease of DiBAC_4_(3) fluorescence (Figure 4B).

### Membrane Depolarization and Loss of Permeability Are Linked in *D. hansenii*

**FIGURE 5:**
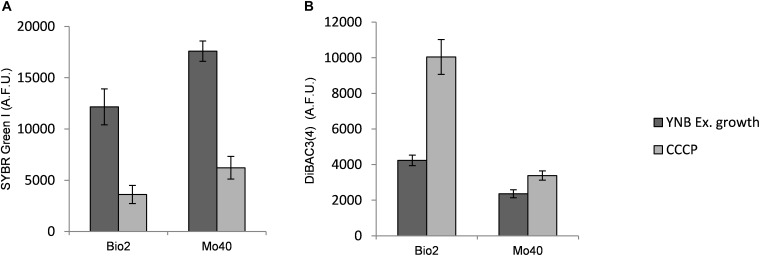
Quantitative analysis of **(A)** SYBR Green I and **(B)** DiBAC4(3) fluorescences detected by FMC after exposure to carbonyl cyanide 3-chlorophenylhydrazone (CCCP), a protonophore inducing membrane depolarization. AFU, Arbitrary Fluorescence Units.

The results obtained suggested that the cultivation under hyper-osmotic conditions induced in Bio2 and Mo40 a loss of permeability to cationic dyes like SYBR Green I, as well as a concomitant membrane depolarization. To assess if the two phenomena were correlated, cells growing under control condition (without salts) were exposed to CCCP, a protonophore that causes membrane depolarization (Zahumenský et al., 2017). The cells were then stained with DiBAC_4_(3), to detect membrane depolarization, and with SYBR Green I, to detect loss of permeability. In comparison to the control cells, at higher levels of DiBAC_4_(3) fluorescence (increase of 137% for Bio2 and 43% for Mo40) corresponded lower levels of SYBR Green I fluorescence (decrease of 80% for Bio2 and 65% for Mo40) (Figure 5). In conclusion, these effects (depolarization and impermeability) were similar to those observed when cells growing on control media (without salts) were exposed to salts (Figure 2).

**FIGURE 6:**
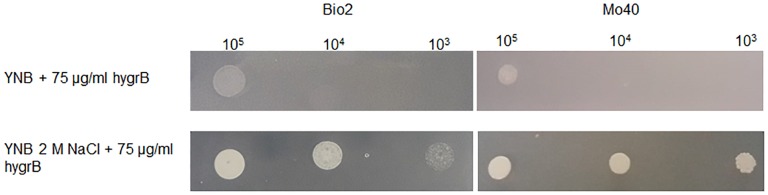
Test for cationic drug resistance. *D. hansenii* cells were spotted on YNB plates supplemented with 75 μg/ml Hygromycin B and containing or not 2 M NaCl. The plates were recovered after 3 days of growth at 30°C.

Membrane potential is known to play a role in the mechanism of cationic drugs toxicity in *S. cerevisiae* (Maresova et al., 2009). Mutants exhibiting hyper-polarization of plasma membranes showed hyper-sensitivity to Hygromycin B (Kodedová and Sychrová, 2015). When *D. hansenii* Bio2 and Mo40 strains were cultivated on solid medium containing 2 M NaCl, they displayed a decreased sensitivity toward this drug (Figure 6), reinforcing the conclusion that the growth under hyper-osmotic conditions alters the membrane permeability to cationic compounds, due to an acquired level of depolarization.

### Membrane Depolarization and Loss of Permeability Occur Also in *S. cerevisiae*

To verify if this correlation occurred also in other less salt tolerant yeast species, we exposed *S. cerevisiae* to hyper-osmotic stress caused by the presence of 0.55 M NaCl. *S. cerevisiae* is known to exhibit maximum tolerance to NaCl 2 M (Lages et al., 1999).

**FIGURE 7:**
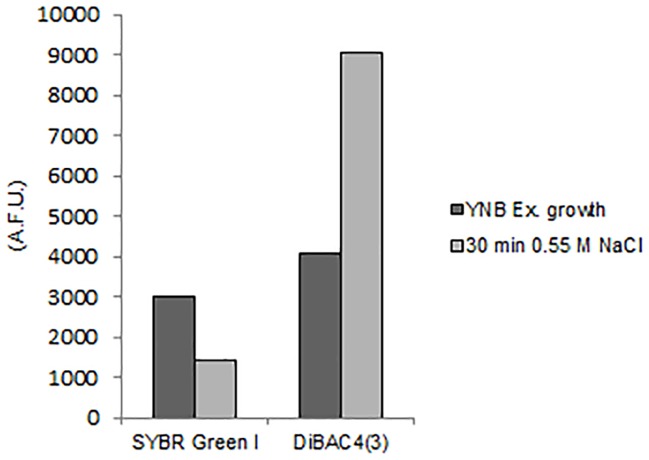
Quantitative analysis of SYBR Green I and DiBAC_4_(3) fluorescences detected by FMC in *S. cerevisiae* cells after exposure to hyper-osmotic stress (0.55 M NaCl). AFU, Arbitrary Fluorescence Units.

The exposition to salts for 30 min triggered membrane depolarization, detected by 122% increase of DiBAC_4_(3) fluorescence in comparison to control cells (Figure 7). Concomitantly the level of SYBR Green I staining decreased of 52% (Figure 7 and Supplementary Table S3).

These results demonstrate that the membrane depolarization induced by hyper-osmotic stress causes a loss of membrane permeability also in *S. cerevisiae*.

## Discussion

In order to develop bioprocesses using seawater or high salt-containing feedstocks, an important point is to analyze their impact on the growth parameters. A screening performed on solid medium containing different concentration of NaCl (range 0.5–2 M) revealed that among yeasts species isolated from endemic animal fauna living at deep-sea hydrothermal vents (Burgaud et al., 2010), *D. hansenii* was the most halotolerant. In this study, we further investigated the halotolerance of two *D. hansenii* strains, Bio2 and Mo40, by carrying out broth cultures in presence or absence of SS 4% (w/v) (containing 0.55 M NaCl) or 2 M NaCl. The presence of SS did not affect the growth in terms of rate and exerted a faint negative effect only on the biomass yield. The presence of higher NaCl concentration (2 M) produces the same effect on the biomass yield. This can reflect a greater energy expenditure to maintain balanced the internal osmotic pressure in presence of hypertonic conditions. The other growth parameters resulted more influenced in presence of 2 M NaCl, with reduction of 42–46% for the growth rate and of 22–24% for the glucose consumption rate (Table 1). Nevertheless, it is noteworthy that for the black yeast *Hortaea werneckii*, which is considered a halotolerant/halophilic species, a growth rate decrease of 60% at salinities between 10 and 17% NaCl on defined minimal medium has been reported (Petrovic et al., 2002). Glycerol was accumulated as a compatible solute to maintain osmotic equilibrium (Figure 1). This response, which can be expected at high salt concentration, as already reported (Gori et al., 2005), was a bit surprising in presence of sea salts, due to the ecological origin of the strains.

By using FCM, we analyzed at single cell level effects related to the hyper-osmotic stress response. The presence of salts in the growth medium causes a reduced cell volume in Bio2 strain, detected in terms of FSC (Supplementary Figure S1). Conversely no significative variations in terms of cell dimension were detectable in Mo40, suggesting that this response to osmotic stress is strain dependent. This effect has been previously described in *S. cerevisiae* and in *D. hansenii*, but using different analytical methods (Prista et al., 1997; Petelenz-Kurdziel et al., 2011). By staining the cells with permeant dyes like SYBR Green I (Figure 2A) and SYTO^TM^ 24 (Supplementary Figure S3), we observed that the cultivation under hyper-osmotic conditions causes a decreased membrane permeability. In addition, we found that the adaptation to hyper-osmotic conditions is strictly associated to the acquisition of plasma membrane depolarization, which is, however, compatible with the ability to grow under these conditions (Figure 1, 2B). These responses are fast, being detectable after 30 min of salt exposure (hyper-osmotic stress) (Figure 3).

By using CCCP as selective proton gradient uncoupling, we demonstrate that membrane depolarization and loss of membrane permeability are strictly associated (Figure 5). Moreover, these responses are not only specifically triggered by salt-generated stress, but seem to be induced also by high concentration of sorbitol (Supplementary Table S2). Our results indicate that the membrane depolarization and the consequent reduced membrane permeabilization can be considered as general osmotic stress responses.

Interestingly, we observed that hyper-osmotic induced membrane depolarization is reversible. Indeed, the elimination of high osmotic pressure causes a rapid restoration of membrane polarization and in turn restores the cellular permeability (Figure 4), underlining how the coordination of these mechanisms is fundamental in the control of cellular homeostasis. Plasma membrane depolarization can then represent another important mechanism contributing to the fast restoration of ions balance by reducing transporter activities. Upon addition of 1 M NaCl, a significant decrease in the abundance of Pma1 H^+^-ATPase, which is the main system pumping protons out of cells, in the *S. cerevisiae* plasma membrane has been reported (Szopinska et al., 2011). Its decreased activity can result in a relative depolarization, and then in a concomitant decrease in the potential-driven influx of toxic sodium cation. On the other hand, a rapid and sharp rise in cytoplasmic calcium level occurs upon osmotic stress, due to a combination of uptake of extracellular Ca^2+^, release of Ca^2+^ from intracellular stores and its limited vacuolar sequestration (Matsumoto et al., 2002), contributing to the membrane depolarization. Depolarization can change in turn the general properties of the membrane, by a different distribution of the plasma membrane components (Grossmann et al., 2007; Herman et al., 2015). Changes in membrane packing related to more disordered structure and sterol re-localization can make depolarized membrane less accessible, and this could play a significant role in fast cellular responses to acute stress conditions. In accordance with these observations, the plasma membrane of depolarized *S. cerevisiae* cells is less sensitive to detergents and cationic drugs (Grossmann et al., 2007; Maresova et al., 2009). By using this approach, we show that the presence of 2 M NaCl increases the resistance of *D. hansenii* to cationic drugs like Hygromycin B (Figure 6).

We describe that these osmotic stress responses are not species-specific but occur also in the less osmotolerant species *S. cerevisiae* (Figure 7). We can then speculate that similar mechanisms can be operative in *D. hansenii* as well as in *S. cerevisiae*, resulting in membrane depolarization and in strongly reduced permeability to cationic compounds like SYBR Green I and Hygromycin B.

In addition, we show that permeant dyes like SYBR Green and SYTO^TM^ 24 can be used to evaluate membrane permeability by FCM. In this respect, it is noteworthy that depending on the physiological conditions, the usage of permeant dyes can be problematic. We reasoned in fact that being well known that response to osmotic stress is very fast (Hohmann, 2015), the conditions employed during the staining (taking approximately 30 min) could affect the cells status. In order to maintain the cells during the staining procedure under the same osmotic pressure present during their growth, avoiding hypo-osmotic shock, cells were stained in their respective growth media. When cells growing under hyper-osmotic condition were stained using hypo-osmotic conditions, like PBS or medium without salt (Supplementary Figure S4 and Figure 4), we obtained different results. Due to the reversibility of some cellular responses, like membrane depolarization and altered permeability, the staining conditions are in fact very relevant, and this point to the importance of optimizing the staining procedures in order to obtain correct results and to avoid misinterpretations (Davey, 2011).

Yeasts are exposed to variations of osmotic pressure during biotechnological processes, and such changes are known to play important roles on cellular viability and metabolic activities, by challenging the integrity of cells. The deep knowledge of these aspects is very relevant for the optimization of industrial bioprocesses, particularly in the case of fermentations/bioconversions performed on high nutrients and/or seawater-based media. An efficient transport of nutrients/substrates and products is in fact at the basis of an efficient cellular performance, and this is the essential prerequisite for the efficiency of the whole industrial process. Moreover, these aspects can be also important for the development of new probiotic strains able to survive to bile salts in the gastrointestinal tract.

## Author Contributions

ClC, SD, BG, and IS performed the experiments. SA performed FCM analysis. CC, ClC, and SA conceived and designed the experiments. CC wrote the manuscript. ClC and SA revised the manuscript.

## Conflict of Interest Statement

The authors declare that the research was conducted in the absence of any commercial or financial relationships that could be construed as a potential conflict of interest.

## Footnotes

1 http://www.univ-brest.fr/ubocc
