# Supplementary material for: Hyper-Osmotic Stress Elicits Membrane Depolarization and Decreased Permeability in Halotolerant Marine Debaryomyces hansenii Strains and in Saccharomyces cerevisiae
Source: Front Microbiol. 2019 Jan 29;10:64. doi: 10.3389/fmicb.2019.00064 (PMC6362939; doi:10.3389/fmicb.2019.00064)
Supplement: Supplementary file 1 [file Table_1.docx]

Supplementary Material

**Hyper-osmotic stress elicits membrane depolarization and decreased permeability in halotolerant marine *Debaryomyces hansenii* strains and in *Saccharomyces cerevisiae.***

**Claudia Capusoni, Stefania Arioli, Silvia Donzella, Benedetta Guidi, Immacolata Serra, Concetta Compagno***

Department of Food, Environmental and Nutritional Sciences, University of Milan, Italy

*** Correspondence:**Concetta Compagno
concetta.compagno@unimi.it

**Supplementary Table S1.** Screening of yeast growth on YNB solid medium containing different concentration of NaCl.

| **Time (h)** | **NaCl (%)** | | **Yeast Strains** | | | | | | | | | | |  | |  |  |  |  | |
| --- | --- | --- | --- | --- | --- | --- | --- | --- | --- | --- | --- | --- | --- | --- | --- | --- | --- | --- | --- | --- |
|  |  |  | **Mo40** | | **Mo 29** | **Mo35** | **Mo38** | **Mo39** | | **Ex7** | **Ex15** | **Bio1** | **Bio2** | **Mo36** | | **Mo34** | **Mo31** | **Mo30** | **Mo22** | |
| 24 | | 0 | ** | | ** | ** | ** | ** | | ** | *** | *** | ** | * | |  | * |  |  | |
|  | | 3 | * | | * | * | * | * | | * | ** | ** | ** |  | |  | * |  |  | |
|  | | 6 | * | |  |  |  | * | |  | * |  | ** |  | |  |  |  |  | |
|  | | 9 | * | |  |  |  |  | |  |  |  | * |  | |  |  |  |  | |
|  | | 12 |  | |  |  |  |  | |  |  |  |  |  | |  |  |  |  | |
| 48 | | 0 | **** | | **** | **** | **** | **** | | **** | **** | **** | **** | *** | | * | **** |  | ** | |
|  | | 3 | **** | | **** | **** | **** | **** | | **** | **** | **** | **** | *** | | * | **** |  | ** | |
|  | | 6 | **** | | *** | **** | *** | *** | | *** | **** | **** | **** | * | | * | **** |  |  | |
|  | | 9 | *** | |  | * |  | *** | | * | ** | ** | ** |  | |  | ** |  |  | |
|  | | 12 |  | |  |  |  | * | |  |  |  | * |  | |  |  |  |  | |
| 120 | | 0 | **** | | **** | **** | **** | **** | | **** | **** | **** | **** | **** | | *** | **** |  | **** | |
|  | | 3 | **** | | **** | **** | **** | **** | | **** | **** | **** | **** | **** | | **** | **** | * | **** | |
|  | | 6 | **** | | **** | **** | **** | **** | | **** | **** | **** | **** | **** | | **** | **** | ** | *** | |
|  | | 9 | **** | | *** | **** | *** | **** | | **** | **** | **** | **** | *** | | *** | **** | ** |  | |
|  | | 12 | **** | |  |  | * | **** | |  | **** | ** | **** |  | | ** | **** |  |  | |
| Abbreviations | | | |  | | | | |  | | | | | |  | | | | |  |
| **Name** | | | | **Genus** | | | | | **Species** | | | | | | **ID number** | | | | |  |
| Bio1 | | | | *Candida* | | | | | *viswanathii* | | | | | | UBOCC-A-208001 | | | | |  |
| Bio2 | | | | *Debaryomyces* | | | | | *hansenii* | | | | | | UBOCC-A-208002 | | | | |  |
| Ex15 | | | | *Pichia* | | | | | *guilliermondii* | | | | | | UBOCC-A-208004 | | | | |  |
| Ex7 | | | | *Rhodotorula* | | | | | *mucilaginosa* | | | | | | UBOCC-A-208010 | | | | |  |
| Mo22 | | | | *Sporobolomyces* | | | | | *roseus* | | | | | | UBOCC-A-208018 | | | | |  |
| Mo29 | | | | *Cryptococcus* | | | | | sp | | | | | | UBOCC-A-208024 | | | | |  |
| Mo30 | | | | *Phaeotheca* | | | | | *triangularis* | | | | | | UBOCC-A-208025 | | | | |  |
| Mo31 | | | | *Candida* | | | | | *atlantica* | | | | | | UBOCC-A-208026 | | | | |  |
| Mo34 | | | | *Hortaea* | | | | | *werneckii* | | | | | | UBOCC-A-208029 | | | | |  |
| Mo35 | | | | *Rhodotorula* | | | | | *mucilaginosa* | | | | | | UBOCC-A-208030 | | | | |  |
| Mo36 | | | | *Leucosporidium* | | | | | *scottii* | | | | | | UBOCC-A-208031 | | | | |  |
| Mo38 | | | | *Rhodosporidium* | | | | | *diobovatum* | | | | | | UBOCC-A-208033 | | | | |  |
| Mo39 | | | | *Candida* | | | | | *marinus* | | | | | | UBOCC-A-208034 | | | | |  |
| Mo40 | | | | *Debaryomyces* | | | | | *hansenii* | | | | | | UBOCC-A-208035 | | | | |  |

**Table S2.** SYBR Green I and DiBAC_4_(3) fluorescences (Arbitrary fluorescence units – AFU) detected by FMC in FL1 channel in *D. hansenii* cells collected under different conditions.

|  | **Bio2** | |  | **Mo40** | |
| --- | --- | --- | --- | --- | --- |
|  | SYBR Green I | DiBAC_4_(3) |  | SYBR Green I | DiBAC_4_(3) |
| **Exponential growth** |  |  |  |  |  |
| YNB | 12152 ± 1725 | 4236 ± 294 |  | 17595 ± 996 | 2362 ± 225 |
| YNB SS | 2866 ± 556 | 7834 ± 343 |  | 7134 ± 1766 | 3426 ± 285 |
| YNB 2 M NaCl | 1452 ± 203 | 9071 ± 526 |  | 7726 ± 760 | 5583 ± 703 |
|  |  |  |  |  |  |
| **Hyper-osmotic stress** | SYBR Green I | DiBAC_4_(3) |  | SYBR Green I | DiBAC_4_(3) |
| YNB exp growth | 12152 ± 1752 | 4236 ± 294 |  | 17595 ± 996 | 2362 ± 225 |
| 30 min YNB SS | 3081 ± 508 | 5853 ± 500 |  | 7355 ± 1134 | 4328 ± 373 |
| 30 min YNB 2 M NaCl | 2647 ± 225 | 8495 ± 256 |  | 10535 ± 619 | 6621 ± 392 |
| CCCP | 3619 ± 855 | 10043 ± 979 |  | 6217 ± 1107 | 3383 ± 259 |
| 2h YNB 2 M sorbitol | 1895 ± 236 | 10835 ± 563 |  | nd | nd |
|  |  |  |  |  |  |
| **Hypo-osmotic stress** | SYBR Green I | DiBAC_4_(3) |  | SYBR Green I | DiBAC_4_(3) |
| YNB 2 M NaCl exp. growth | 1452 ± 203 | 9071 ± 526 |  | 7726 ± 760 | 4999 ± 703 |
| 30 min on YNB | 4618 ± 448 | 2915 ± 288 |  | 10568 ± 1442 | 2658 ± 438 |

**Table S3.** SYBR Green I and DiBAC_4_(3) fluorescences (Arbitrary fluorescence units – AFU) detected by FMC in FL1 channel in *S.cerevisiae* cells collected under hyper-osmotic stress.

|  | SYBR Green I | DiBAC_4_(3) |
| --- | --- | --- |
| YNB exp. growth | 2985 ± 284 | 4064 ± 332 |
| 30 min YNB 0.55 M NaCl | 1430 ± 146 | 9028 ± 772 |

**Figure S1.** FCM analysis showing Bio2 and Mo40 cell size (as FSC) under different growth conditions. Blue line: cells exponentially growing on YNB (control cells); green line: cells growing in presence of SS; red line: cells growing in presence of 2 M NaCl.

**Figure S2.** FCM analysis showing SYBR Green I fluorescences detected in FL1 channel. **(A)** Bio2 cells stained after growth on YNB (control cells); **(B)** in presence of SS; **(C)** in presence of 2 M NaCl; **(D)** Mo40 cells stained after growth on YNB (control cells); **(E)** in presence of SS; **(F)** in presence of 2 M NaCl.


**Figure S3.** FMC analysis showing SYTO™ 24 fluorescence detected in FL1 channel in Bio2 cells stained after growth (A) on YNB (control cells); (B) on YNB plus SS; (C) on YNB plus 2 M NaCl.

**Figure S4.** FMC analysis showing SYBR Green I fluorescence detected in FL1 channel in Bio2 cells after permeabilization with ethanol 70 % (v/v) or in cells stained in PBS after growth on YNB (control cells) **(**A, D**)**, on YNB plus SS (B, E), on YNB plus 2 M NaCl (C, F).

**Figure S5**. cFSE fluorescence detected in FL1 channel in Bio2 cells collected (A) after growth on YNB (control cells); (B) on YNB plus SS; (C) on YNB plus 2 M NaCl, and in Mo40 cells (D) grown on YNB (control cells); (E) grown on YNB plus SS; (F) grown on YNB plus 2 M NaCl.
